# Viscoelastic Methods of Blood Clotting Assessment – A Multidisciplinary Review

**DOI:** 10.3389/fmed.2015.00062

**Published:** 2015-09-14

**Authors:** Jan Benes, Jan Zatloukal, Jakub Kletecka

**Affiliations:** ^1^Department of Anesthesiology and Intensive Care Medicine, Faculty of Medicine and Teaching Hospital in Plzen, Charles University in Prague, Plzen, Czech Republic; ^2^Faculty of Medicine in Plzen, Biomedical Centre, Charles University in Prague, Plzen, Czech Republic

**Keywords:** coagulation, viscoelastic methods, point-of-care testing, trauma-induced coagulopathy, transfusion, thrombosis

## Abstract

Viscoelastic methods (VEM) made available the bedside assessment of blood clotting. Unlike standard laboratory tests, the results are based on the whole blood coagulation and are available in real time at a much faster turnaround time. In combination with our new knowledge about pathophysiology of the trauma-induced coagulopathy, the goal-oriented treatment protocols have been recently proposed for the initial management of bleeding in trauma victims. Additionally, the utility of viscoelastic monitoring devices has been proved even outside this setting in cardiosurgical patients or those undergoing liver transplantation. Many other situations were described in literature showing the potential use of bedside analysis of coagulation for the management of bleeding or critically ill patients. In the near future, we may expect further improvement in current bedside diagnostic tools enabling not only the assessment of secondary hemostasis but also the platelet aggregation. More sensitive assays for new anticoagulants are underway. Aim of this review is to offer the reader a multidisciplinary overview of VEM and their potential use in anesthesiology and critical care.

## Introduction

In the contemporary emergency, intensive care medicine and anesthesia time are regarded as one of the most important factors affecting the patients’ outcome. Timely administration of antibiotics affects septic patients’ survival; prompt volume and/or catecholamine resuscitation have been repeatedly associated with better outcomes in many different critical states. On the other hand, individualization and goal-oriented treatments also seem to offer this advantage. In order to be able to tailor the treatment to patients’ individual needs, the information describing the actual state is paramount. Because the nature of acute critical illness is often changing in very rapid pace, many contemporary diagnostic tools are too slow and hence inappropriate to combine these two approaches of timely administered but goal-oriented treatments. For this reason, several point-of-care testing (POCT) devices were introduced into the clinical praxis in the last few years or decades.

Unlike others (hemoglobin or blood glucose bedside analyzers), the POCT as a tool for assessing blood clotting has been disregarded for quite a long time. The history of viscoelastic methods (VEM) has started already in the beginning of twentieth century, and the first prototype of thromboelastography (TEG) was introduced in 1948 by Harter (1). Nevertheless, the analysis of activated clotting time with the use of Hemochron or similar devices in patients on heparin anticoagulation was for a long time the only widely applied clotting POCT in clinical praxis. The paradigm shift based on the new knowledge about massive bleeding and trauma-associated coagulopathies observed in the late 1990s and in the beginning of twenty-first century marked the increased interest in VEM. Nowadays, the TEG/thromboelastometry seems to be a very important and rapidly developing field of acute medicine. Its ability to help to distinguish the most important coagulation deficiencies makes it increasingly interesting in the goal-oriented coagulation management of massive bleeding. Especially in view of the fact that standard laboratory coagulation assays (thrombin and prothrombin time and activated partial thromboplastin time) have been shown as insufficient to correlate with the bleeding in acute trauma setting (2). Also, the turnaround times of standard tests were demonstrated to be about 45 min in comparison with 10 min when using the first applicable VEM-derived variables (3, 4). Besides the initial management of bleeding in traumatized or operated patients, the evidence starts to accumulate the fact that VEM could also offer important information in the later course of critical illness to diagnose and manage the hypercoagulative phenotype (5).

## Widely Available Methods of VEM

**Figure 1:**
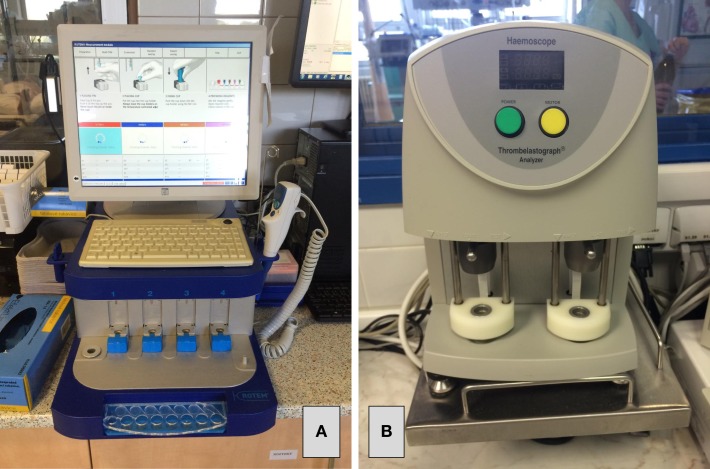
**The most commonly used viscoelastic devices – the ROTEM^®^ device (A) and the TEG^®^ device (B)**.

In the contemporary praxis, two devices are mostly used: the older TEG^®^ device (Haemoscope Corporation, Niles, IL, USA) and newer ROTEM^®^ (Pentapharm GmbH, Munich, Germany) – see Figure 1. Third device, namely the Sonoclot Analyzer (Sienco Inc., Arvada, CO, USA), is also available, whereas there is much less evidence for the use of this third device in clinical praxis than the previous ones. Principles of all the VEM devices are based on the measurement of change in viscoelastic properties of the whole blood during the clot formation. The firmer the clot, the higher is the force opposing the movement of rotating (TEG^®^ and ROTEM^®^) or vibrating (Sonoclot) particles of the measuring device. Unlike laboratory methods of blood coagulation, the VEM devices are not only able to assess the time needed to form fibrin polymers, but they also allow the monitoring of further clot formation, its strength, and in some cases the potential lysis as well. The use of the whole blood enables to evaluate the influence of blood cellular components and their phospholipid surfaces on clot formation and its final strength. The tests are mostly performed in heated cup that allows the clot formation assessment under the real conditions of patients’ body temperature. The detailed methodology of all three devices is given in multiple published papers (6, 7).

**Figure 2:**
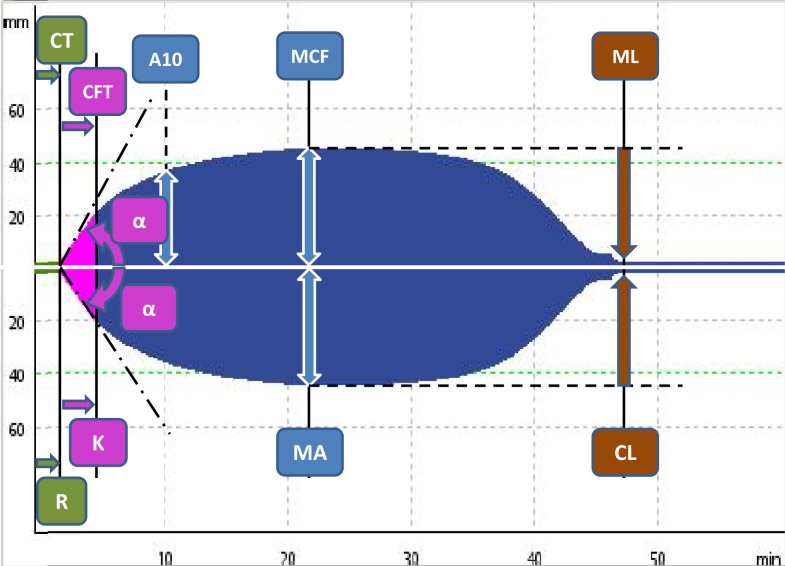
**The typical tracings of ROTEM^®^ (upper panel) and TEG^®^ devices (lower panel) with the most prominent parameters of both methods with the comparison (see also Table 2)**.

In routine praxis, several clotting activators and cofactors are used to evaluate different coagulation pathways. Both TEG^®^ and ROTEM^®^ influencing each other’s development offer similar assays (Table 1). Even though similarities in parameters offered by these methods can be found, the numerical values are not directly interchangeable due to different methods of assessment of viscoelastic forces and time-wise definition of variables (Table 2; Figure 2). Additionally, these widely used variables and also other derived or calculated parameters are displayed in the devices, and some new variables are under development (8). In a similar method, several specific assays are described in the literature further broadening the analytical possibilities of this method (9, 10).

**Table 1:** **List of commercially available tests for ROTEM^®^ and TEG^®^ devices**.

Test	Activator	Description
**ROTEM^®^ TESTS**		
NATEM	None	Clot analysis of native blood
EXTEM	Tissue factor	Test of “extrinsic pathway” – fastest clot analysis; usable in comparison with APTEM and FIBTEM
INTEM	Contact activator	Test of “intrinsic pathway”; usable in comparison with HEPTEM
FIBTEM	Tissue factor + cytochalasin D	Test of fibrin net polymerization after platelet inhibition (in comparison with EXTEM)
APTEM	Tissue factor + aprotinin	Test of fibrinolysis (in comparison with EXTEM)
HEPTEM	Contact activator + heparinase	Test of residual heparinization (in comparison with INTEM)
**TEG^®^ TESTS**		
Na-TEG	None	Clot analysis of native blood
RapidTEG	Kaolin + tissue factor	Test of both “intrinsic and extrinsic pathways”
Kaolin TEG	Kaolin	Test of “intrinsic pathway”
Kaolin TEG with heparinase	Kaolin + heparinase	Test of residual heparinization (in comparison with Kaolin TEG)
Functional fibrinogen	Kaolin + GpIIb/IIIa inhibition	Test of fibrin net polymerization after platelet inhibition (in comparison with Kaolin TEG)

**Table 2:** **List and comparison of the most important variables describing the VEM-derived curve**.

Variable	ROTEM^®^	TEG^®^
Clotting time (2 mm amplitude)	CT (clotting time)	R (reaction time)
	Normal (EXTEM) = 42–74 s	Normal (citrate/kaolin) = 3–8 min
	Normal (INTEM) = 137–246 s	
Clot formation/kinetics (20 mm amplitude)	CFT (clot formation time)	K (kinetics)
	Normal (EXTEM) = 46–148 s	Normal (citrate/kaolin) = 1–3 min
	Normal (INTEM) = 40–100 s	
Clot strengthening (angle of clot formation)	Alfa angle (slope of tangent at 2 mm amplitude)	Alfa angle (slope between r and k points)
	Normal (EXTEM) = 63–81°	Normal (citrate/kaolin) = 55–78°
	Normal (INTEM) = 71–82°	
Amplitude/maximal firmness	MCF (maximum clot firmness)	MA (maximal amplitude)
	Normal (EXTEM) = 49–71 mm	Normal (citrate/kaolin) = 51–69 mm
	Normal (INTEM) = 52–72 mm	
	Normal (FIBTEM) = 9–25 mm	
	A5, A10, etc. – amplitudes at dedicated time-points predicting the final clot firmness
Lysis	LI30, LI60, ML	CL30, CL60, CL

## VEM in Acute Trauma Care

Till now, TIC was believed to be caused by the consumption of coagulation factors in conjunction with the so-called lethal triad (hypothermia, acidosis, and dilution). In order to reverse the consumption and dilution-limited crystalloid infusions and the use of massive transfusion protocols with 1:1:1 proportion of fresh-frozen plasma, platelet concentrates and erythrocytes (sometimes termed “hemostatic” or “whole blood” resuscitation) were proposed (11). In more than 30 studies (mostly retrospective or observational), this approach was associated with improved survival (even after the survival bias being acknowledged) (12, 13), and it is currently widely adopted in North America (14). However, in recently published observations, even this aggressive high ratio of blood component therapy (1:1 fresh-frozen plasma to packed red blood cells) was unable to reverse the TIC (15, 16). Moreover, massive transfusion protocols expose patients to risks associated with transfusion, including immune reactions or transfusion-related acute lung injury (17).

In the recent years, several important findings were published altering the simplistic “consumption/dilution” view on TIC (18, 19). Rourke et al. (20) described the association of TIC with low levels of fibrinogen hypothesizing that the consumption of fibrinogen occurs in much more faster pace than that of other factors in trauma bleeding. Actually, only limited number of trauma patients (about 20%) experience severe deficiency of clotting factors, namely factor V (21). Few studies verified this finding of trauma-associated hypofibrinogenemia (22, 23). Faster turnaround of fibrinogen associated with hyperfibrinolysis is another reason for hypofibrinogenemia in some patients. Direct tissue trauma, organ hypoperfusion (24), endothelial disruption (25), or protein C activation was found to contribute for increased plasmin formation and fibrin degradation (26–28). The real proportion of patients experiencing trauma-associated hyperfibrinolysis is unknown. Severe hyperfibrinolysis defined as more than 15% lysis observed within 30 min after reaching maximal clot amplitude was described in more than 5% of patients and associated with 80% fatality rate (Table 3). But even fibrinolysis of much milder extent (3% lysis in 30 min derived from TEG^®^ measurement) was associated with increased transfusion needs and higher mortality (29). If the survival benefit of antifibrinolytics is observed in the recent large multi-centric CRASH-2 trial, one may hypothesize that hyperfibrinolysis occurs much more frequently than previously thought. These findings promoted the use of fibrinogen concentrates as well as the use of antifibrinolytic agents in routine praxis.

**Table 3:** **The occurrence of trauma-associated hyperfibrinolysis**.

	Population	Hyperfibrinolysis incidence	Mortality
Levrat (30)	87	6%	100%
Caroll (31)	161	2.5%	67%
Schochl (32)	Not available	33 pts	88%
Tauber (33)	334	6.8%	85.7%
Kashuk (34)	61	18%	64%
Global population	643	6.7%	80.9%

**Figure 3:**
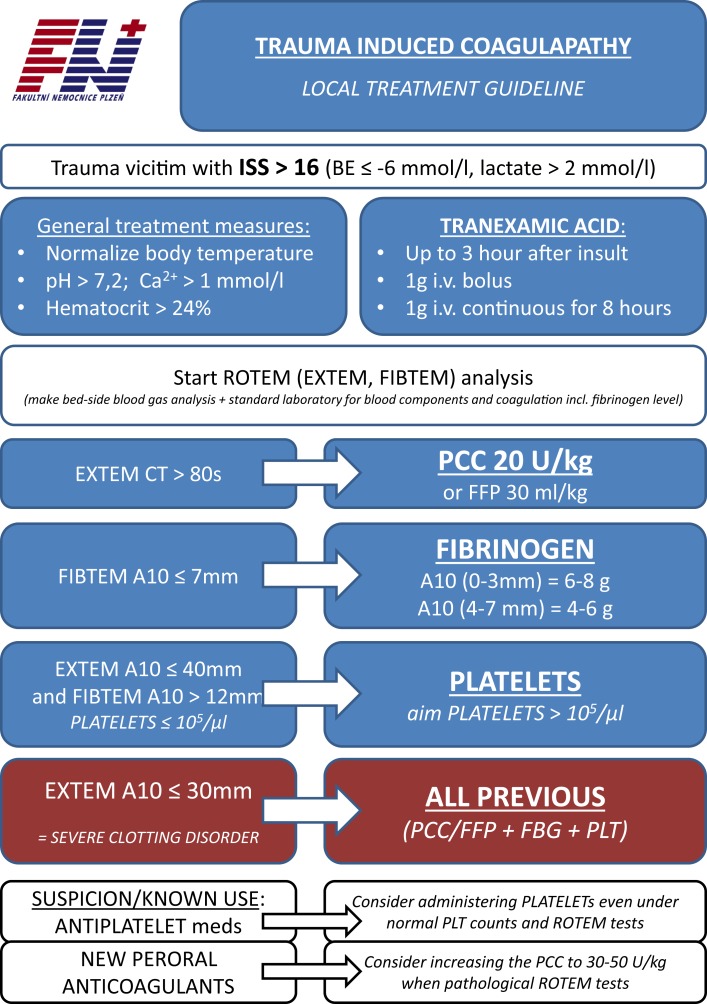
**The protocol of acute traumatic coagulopathy of the authors’ institution (English translation)**.

Viscoelastic methods helped to delineate these most prominent features of what we call nowadays acute trauma coagulopathy. Both low fibrinogen levels and fibrinolysis are diagnosable with the use of VEM-specific tests in real time. Hence, these methods can substantially contribute to the management of acute trauma-associated bleeding (35). Several goal-oriented protocols are available in literature helping to couple the VEM test results with proper treatment (36) (see also Figure 3 for authors’ own institution protocol). The early administration of tranexamic acid (sometimes even in the prehospital setting) and goal-oriented fibrinogen or supplementation of coagulation factors decreased the use of blood products, namely fresh-frozen plasma. The so-called plasma-free approach, in which factor concentrates are only used for coagulation management, has evolved in some institutions in the extreme. This approach may further decrease the risks of blood products therapy. However, the goal-oriented coagulation management and plasma-free approach in trauma victims are still a matter of debate. Some observation studies have demonstrated the significant drop in use of blood products (37–40), but no prospective randomized trial compared the plasma-free VEM-goal-oriented treatment with the massive transfusion protocols (41). Nevertheless, in view of this evidence, the use of VEM as a guide of bleeding management in trauma victims was upgraded to “1C” in the most recent European Trauma Treatment Guidelines (42).

## VEM for Perioperative Care

Unlike the use of VEM in acute trauma care, the utility of these methods for managing acute bleeding in cardiac or hepatic surgery is much more established in the literature. Both named as clinical scenarios are often coupled with the coagulopathy of complex origin, which may be very difficult to assess using standard laboratory test. In patients undergoing cardiac bypass surgery, several factors can impede blood clotting making the management very demanding: the exposition of blood components to the extracorporeal circuit, possible coagulation factors dilution and/or consumption, use of heparin or other anticoagulants, and finally chronic antiplatelet mediation (43). VEM assays able to eliminate heparin influence (HEPTEM or Kaolin/HeparinaseTEG) may help to distinguish between residual heparinization and other sources of bleeding. In a similar way, blood clotting in patients undergoing orthotopic liver transplantation or large liver resection may be altered by chronic liver disease with the malproduction of pro- and anti-coagulant factors not only by major blood loss and postoperative acute liver failure but also by the fibrinolytic shutdown and procoagulant state (44). VEM devices are not able to elucidate all of these problems (see Limitations of Contemporary VEM Analyses) but, unlike standard methods, may better assess the dynamic balance of the secondary hemostasis and lysis (45).

Several studies were published in recent years using TEG^®^ or ROTEM^®^ driven protocols of care especially in cardiosurgical procedures. Wikkelsoe’s Cochrane review group (46) managed to identify nine randomized trials (eight cardiosurgical and one orthotopic liver transplantation) showing the overall benefit of VEM-managed patients in surrogate markers like bleeding or number of used blood products. More recently, Weber et al. (47) showed significant decrease not only in the use of blood products or bleeding risk but also in 6-month mortality and demonstrated overall economic benefit. The National Institute for Health and Care Excellence recommends in its recent guidelines that TEG^®^ and ROTEM^®^ devices may be used to help monitor blood clotting during and after the heart surgery (48).

There is a sparse evidence of using the VEM for other procedures associated with the risk of major bleeding (i.e., obstetric). During pregnancy, the coagulation system changes in a very complex way meeting its peak at the moment of delivery. Unlike other situations, the postpartum hemorrhage is often associated with both increased (and disseminated) thrombi generation and bleeding due to the consumption of coagulation factors and the fibrin lysis. The use of VEM can help significantly in the nonsurgical management of severe postpartum hemorrhage (49); however, this proposal is built on assumptions based on acute trauma or cardiosurgical care.

## Perspectives of VEM Analysis

The utility of VEM for management of bleeding has significantly expanded the knowledge and also the use of these devices among the anesthesiologists and the critical care physicians. However, it seems that the applicability reaches much further than the initial trauma or perioperative bleeding risk assessment. Several authors have studied the utility of VEM for the assessment of sepsis-associated coagulopathy (50, 51). The association of VEM diagnosed hypocoagulability with the severity of disease, and mortality was evidenced in recent systematic review (52). However, having only low number of relevant studies and rather complex nature of sepsis-associated coagulopathy, possibly more data are needed before the routine use of VEM for this indication.

In the recent years, several authors have demonstrated that VEM are able to assess not only the hypocoagulable phenotype but also the hypercoagulable phenotype, which develops as a contra regulatory mechanism several hours or days after traumatic or surgical injury (53). Hypercoagulability assessed using the standard VEM variables (clotting time, initial angle, or maximal firmness) was demonstrated to be associated with the rate of thromboembolic events in the later course of illness (5, 54). Park et al. (55) demonstrated that trauma patients with subsequent pulmonary embolization had increased angle and amplitude of clot formation within first 48 h as compared with the control group, but no difference in standard coagulation tests was observed. Recently, Müller et al. (56) used VEM tests (total clot strength – G) to separate patients with multiple trauma into those having hypo-, normo-, and hypercoagulable states. A hypercoagulable profile (G ≥ 11.7 dynes cm^−2^) was observed in 10% of trauma population. Using novel parameters obtainable from the VEM curve, Gonzalez et al. (8) were able to separate the platelet and enzymatic hypercoagulability. This new insight enabled by VEM to monitor the hypercoagulable phenotype may significantly affect the way how we would possibly prescribe thromboprophylaxis for the years to come. It was concluded by Patel (57) that most thromboembolic events occurred not only because the thromboprophylaxis was not prescribed, but also because it was not effective. The use of VEM not only for goal-directed bleeding management but also for the thromboprophylaxis seems to be alluring; however, nowadays we are lacking data for such approach. Definitely, before the routine application of VEM for thromboprophylaxis management, more specific tests for the assessment of anticoagulants use and their effectivity (see Limitations of Contemporary VEM Analyses) and possibly also new variables are needed.

## Limitations of Contemporary VEM Analyses

The coagulation assessment of VEM using full blood is much more complex than the standard laboratory tests. Unlike standard methods performed under optimal conditions (often 37°C), most VEM devices are able to adapt to the actual body temperature. However, some aspects of *in vivo* clotting are immeasurable by this method. First, most of contemporary practiced VEM assays use decalcinated blood for practical reason; hence, calcium levels have to be tested separately. The historical TEG method used native blood, where the NATEM also enables to alleviate this, but the use of native (calcinated) blood cannot account for the time between clotting commencement (blood taking) and initiation of the exam. More importantly, all the VEM tests can only assess the secondary hemostasis. Given the cellular theory of hemostasis, the influence of endothelium–platelet interaction (primary hemostasis) cannot be examined. Because most of the generally used antiaggregant medication influences primary hemostasis either by affecting the cyclooxygenase-1 (aspirin or other nonsteroidal anti-inflammatory drugs) or by blocking the adenosine-di-phosphate receptor (clopidogrel and similar substances), current VEM tests are blind to their effect. The ROTEM platelet^®^ platform introduced recently combines the VEM with two-channel multiplate aggregometry enabling to assess the effect of common antiplatelet medication. Similarly, the “Platelet Mapping” TEG modality uses ADP + arachidonic acid to stimulate and analyze platelet aggregation. Use and effect of such POCT for clinical routine were not tested yet.

Furthermore, the influence of new anticoagulants (direct thrombin inhibitors – dabigatran, argatroban; direct factor Xa inhibitors – rivaroxaban, apixaban) as well as of low-molecular weight heparins (specifically anti-Xa activity) on VEM assays is not described well in the literature. All these anticoagulants affect the secondary hemostasis, which should make them evaluable by the VEM tests. However, Schaden et al. (10) demonstrated limited correlation between clotting time values and anti-Xa activity measured by classical INTEM test, which improved when specific tests (PiCT – prothrombinase-induced clotting or LowTF – low-tissue factor activated modification) were applied. Eller et al. (58) demonstrated that ROTEM^®^ assessed clotting time was able to assess the *in vitro* therapeutic concentrations of most of the tested novel per oral anticoagulants (but fondaparinux). However, the prolongation of clotting time is a very unspecific marker that may delay time to proper treatment in case of need. Ecarin modification of ROTEM tests was used by Schaden (9) to improve the significance toward argatroban recently. Whether these novel modifications will help in elucidating the effect of other novel anticoagulants is still unresolved. Moreover, these more specific tests are not clinically available and therefore not routinely performed. This makes the assessment of anticoagulation via VEM tests elusive.

## Conclusion

Viscoelastic methods of coagulation assessment offer time-relevant information about the function of secondary hemostasis. Based on their assessment, a goal-oriented treatment of trauma-associated coagulopathy is feasible decreasing the exposition to blood products and associated risks. In patients undergoing cardiac or liver surgery, the use of VEM was found to improve care, and similar findings may be also expected from the other major bleeding scenarios. The use of VEM in other situations (i.e., septic coagulopathy, hypercoagulation assessment) might be enabled in the near future. But as with any other monitoring tools, it is not the device to blame or honor. The VEM can affect patients’ outcomes only when we will use them and find treatments based on the derived variables affecting patients’ outcome, which should be the agenda of our research activities for the next few years.

## Authors Contribution

JB wrote the main body of the manuscript, and JZ and JK significantly participated on the literature review and critically reviewed the manuscript; all the authors approved the final form of the text.

## Conflict of Interest Statement

The authors declare that the research was conducted in the absence of any commercial or financial relationships that could be construed as a potential conflict of interest.

## Abbreviations

ATC, acute trauma coagulopathy; POCT, point-of-care testing; TIC, trauma-induced coagulopathy; VEM, viscoelastic methods.

## References

[1] Hartert H Blutgerinnungsstudien mit der Thrombelastographie, einem neuen Untersuchungsverfahren. Klin Wochenschr (1948) 26(37):577–83. 10.1007/BF01697545 18101974

[2] Lier H Böttiger BW Hinkelbein J Krep H Bernhard M. Coagulation management in multiple trauma: a systematic review. Intensive Care Med (2011) 37(4):572–82. 10.1007/s00134-011-2139-y 21318436

[3] Woolley T Midwinter M Spencer P Watts S Doran C Kirkman E. Utility of interim ROTEM(^®^) values of clot strength, A5 and A10, in predicting final assessment of coagulation status in severely injured battle patients. Injury (2013) 44(5):593–9. 10.1016/j.injury.2012.03.018 22487164

[4] Davenport R Manson J De’Ath H Platton S Coates A Allard S Functional definition and characterization of acute traumatic coagulopathy. Crit Care Med (2011) 39(12):2652–8. 10.1097/CCM.0b013e3182281af5 21765358 PMC3223409

[5] Hincker A Feit J Sladen RN Wagener G. Rotational thromboelastometry predicts thromboembolic complications after major non-cardiac surgery. Crit Care (2014) 18(5):549. 10.1186/s13054-014-0549-2 25292221 PMC4200117

[6] Ganter MT Hofer CK. Coagulation monitoring: current techniques and clinical use of viscoelastic point-of-care coagulation devices. Anesth Analg (2008) 106(5):1366–75. 10.1213/ane.0b013e318168b367 18420846

[7] Keene DD Nordmann GR Woolley T. Rotational thromboelastometry-guided trauma resuscitation. Curr Opin Crit Care (2013) 19(6):605–12. 10.1097/MCC.0000000000000021 24240827

[8] Gonzalez E Kashuk JL Moore EE Silliman CC. Differentiation of enzymatic from platelet hypercoagulability using the novel thrombelastography parameter delta (delta). J Surg Res (2010) 163(1):96–101. 10.1016/j.jss.2010.03.058 20605586 PMC4373617

[9] Schaden E Schober A Hacker S Kozek-Langenecker S. Ecarin modified rotational thrombelastometry: a point-of-care applicable alternative to monitor the direct thrombin inhibitor argatroban. Wien Klin Wochenschr (2013) 125(5–6):156–9. 10.1007/s00508-013-0327-1 23440521

[10] Schaden E Schober A Hacker S Spiss C Chiari A Kozek-Langenecker S. Determination of enoxaparin with rotational thrombelastometry using the prothrombinase-induced clotting time reagent. Blood Coagul Fibrinolysis (2010) 21(3):256–61. 10.1097/MBC.0b013e328337014c 20087172

[11] Dutton RP. Haemostatic resuscitation. Br J Anaesth (2012) 109(Suppl 1):i39–46. 10.1093/bja/aes389 23242750

[12] Ho AMH Dion PW Yeung JHH Holcomb JB Critchley LAH Ng CSH Prevalence of survivor bias in observational studies on fresh frozen plasma:erythrocyte ratios in trauma requiring massive transfusion. Anesthesiology (2012) 116(3):716–28. 10.1097/ALN.0b013e318245c47b 22270506

[13] Miller TE. New evidence in trauma resuscitation – is 1:1:1 the answer? Perioper Med (Lond) (2013) 2(1):13. 10.1186/2047-0525-2-13 24472306 PMC3964329

[14] Dutton RP. Management of traumatic haemorrhage – the US perspective. Anaesthesia (2015) 70(Suppl 1):108–11,e38. 10.1111/anae.12894 25440404

[15] Khan S Brohi K Chana M Raza I Stanworth S Gaarder C Hemostatic resuscitation is neither hemostatic nor resuscitative in trauma hemorrhage. J Trauma Acute Care Surg (2014) 76(3):561–7. discussion 567, 10.1097/TA.0000000000000146 24553520

[16] Khan S Davenport R Raza I Glasgow S De’Ath HD Johansson PI Damage control resuscitation using blood component therapy in standard doses has a limited effect on coagulopathy during trauma hemorrhage. Intensive Care Med (2015) 41(2):239–47. 10.1007/s00134-014-3584-1 25447807

[17] Seghatchian J Samama MM. Massive transfusion: an overview of the main characteristics and potential risks associated with substances used for correction of a coagulopathy. Transfus Apher Sci (2012) 47(2):235–43. 10.1016/j.transci.2012.06.001 22770808

[18] Frith D Brohi K. The pathophysiology of trauma-induced coagulopathy. Curr Opin Crit Care (2012) 18(6):631–6. 10.1097/MCC.0b013e3283599ab9 23010636

[19] Cap A Hunt B. Acute traumatic coagulopathy. Curr Opin Crit Care (2014) 20(6):638–45. 10.1097/MCC.0000000000000158 25340382

[20] Rourke C Curry N Khan S Taylor R Raza I Davenport R Fibrinogen levels during trauma hemorrhage, response to replacement therapy, and association with patient outcomes. J Thromb Haemost (2012) 10(7):1342–51. 10.1111/j.1538-7836.2012.04752.x 22519961

[21] Rizoli SB Scarpelini S Callum J Nascimento B Mann KG Pinto R Clotting factor deficiency in early trauma-associated coagulopathy. J Trauma (2011) 71(5 Suppl 1):S427–34. 10.1097/TA.0b013e318232e5ab 22071999 PMC3241929

[22] Inaba K Karamanos E Lustenberger T Schöchl H Shulman I Nelson J Impact of fibrinogen levels on outcomes after acute injury in patients requiring a massive transfusion. J Am Coll Surg (2013) 216(2):290–7. 10.1016/j.jamcollsurg.2012.10.017 23211116

[23] Hagemo JS Stanworth S Juffermans NP Brohi K Cohen M Johansson PI Prevalence, predictors and outcome of hypofibrinogenaemia in trauma: a multicentre observational study. Crit Care (2014) 18(2):R52. 10.1186/cc13798 24666991 PMC4056526

[24] Frith D Goslings JC Gaarder C Maegele M Cohen MJ Allard S Definition and drivers of acute traumatic coagulopathy: clinical and experimental investigations. J Thromb Haemost (2010) 8(9):1919–25. 10.1111/j.1538-7836.2010.03945.x 20553376

[25] Johansson PI Stensballe J Rasmussen LS Ostrowski SR. A high admission syndecan-1 level, a marker of endothelial glycocalyx degradation, is associated with inflammation, protein C depletion, fibrinolysis, and increased mortality in trauma patients. Ann Surg (2011) 254(2):194–200. 10.1097/SLA.0b013e318226113d 21772125

[26] Gando S Tedo I Kubota M. Posttrauma coagulation and fibrinolysis. Crit Care Med (1992) 20(5):594–600. 10.1097/00003246-199205000-00009 1533358

[27] Cohen MJ Call M Nelson M Calfee CS Esmon CT Brohi K Critical role of activated protein C in early coagulopathy and later organ failure, infection and death in trauma patients. Ann Surg (2012) 255(2):379–85. 10.1097/SLA.0b013e318235d9e6 22133894 PMC3549308

[28] Schöchl H Voelckel W Maegele M Solomon C. Trauma-associated hyperfibrinolysis. Hamostaseologie (2012) 32(1):22–7. 10.5482/ha-1178 22009115

[29] Chapman MP Moore EE Ramos CR Ghasabyan A Harr JN Chin TL Fibrinolysis greater than 3% is the critical value for initiation of antifibrinolytic therapy. J Trauma Acute Care Surg (2013) 75(6):961–7. discussion 967, 10.1097/TA.0b013e3182aa9c9f 24256667 PMC4072127

[30] Levrat A Gros A Rugeri L Inaba K Floccard B Negrier C Evaluation of rotation thrombelastography for the diagnosis of hyperfibrinolysis in trauma patients. Br J Anaesth (2008) 100(6):792–7. 10.1093/bja/aen083 18440953

[31] Carroll RC Craft RM Langdon RJ Clanton CR Snider CC Wellons DD Early evaluation of acute traumatic coagulopathy by thrombelastography. Transl Res (2009) 154(1):34–9. 10.1016/j.trsl.2009.04.001 19524872

[32] Schöchl H Frietsch T Pavelka M Jámbor C. Hyperfibrinolysis after major trauma: differential diagnosis of lysis patterns and prognostic value of thrombelastometry. J Trauma (2009) 67(1):125–31. 10.1097/TA.0b013e31818b2483 19590321

[33] Tauber H Innerhofer P Breitkopf R Westermann I Beer R El Attal R Prevalence and impact of abnormal ROTEM(R) assays in severe blunt trauma: results of the ‘Diagnosis and Treatment of Trauma-Induced Coagulopathy (DIA-TRE-TIC) study’. Br J Anaesth (2011) 107(3):378–87. 10.1093/bja/aer158 21705350

[34] Kashuk JL Moore EE Sawyer M Wohlauer M Pezold M Barnett C Primary fibrinolysis is integral in the pathogenesis of the acute coagulopathy of trauma. Ann Surg (2010) 252(3):434–42. discussion 443, 10.1097/SLA.0b013e3181f09191 20739843

[35] Spahn DR. TEG^®^- or ROTEM^®^-based individualized goal-directed coagulation algorithms: don’t wait – act now! Crit Care (2014) 18(6):637. 10.1186/s13054-014-0637-3 25672839 PMC4331375

[36] Schöchl H Maegele M Solomon C Görlinger K Voelckel W. Early and individualized goal-directed therapy for trauma-induced coagulopathy. Scand J Trauma Resusc Emerg Med (2012) 20:15. 10.1186/1757-7241-20-15 22364525 PMC3306198

[37] Schöchl H Nienaber U Hofer G Voelckel W Jambor C Scharbert G Goal-directed coagulation management of major trauma patients using thromboelastometry (ROTEM)-guided administration of fibrinogen concentrate and prothrombin complex concentrate. Crit Care (2010) 14(2):R55. 10.1186/cc8948 20374650 PMC2887173

[38] Schöchl H Nienaber U Maegele M Hochleitner G Primavesi F Steitz B Transfusion in trauma: thromboelastometry-guided coagulation factor concentrate-based therapy versus standard fresh frozen plasma-based therapy. Crit Care (2011) 15(2):R83. 10.1186/cc10078 21375741 PMC3219338

[39] Kashuk JL Moore EE Le T Lawrence J Pezold M Johnson JL Noncitrated whole blood is optimal for evaluation of postinjury coagulopathy with point-of-care rapid thrombelastography. J Surg Res (2009) 156(1):133–8. 10.1016/j.jss.2009.03.046 19577246

[40] Nardi G Agostini V Rondinelli B Russo E Bastianini B Bini G Trauma-induced coagulopathy: impact of the early coagulation support protocol on blood product consumption, mortality and costs. Crit Care (2015) 19(1):83. 10.1186/s13054-015-0817-9 25880548 PMC4383211

[41] Da Luz LT Nascimento B Shankarakutty AK Rizoli S Adhikari NK. Effect of thromboelastography (TEG^®^) and rotational thromboelastometry (ROTEM^®^) on diagnosis of coagulopathy, transfusion guidance and mortality in trauma: descriptive systematic review. Crit Care (2014) 18(5):518. 10.1186/s13054-014-0518-9 25261079 PMC4206701

[42] Spahn DR Bouillon B Cerny V Coats TJ Duranteau J Fernández-Mondéjar E Management of bleeding and coagulopathy following major trauma: an updated European guideline. Crit Care (2013) 17(2):R76. 10.1186/cc12685 23601765 PMC4056078

[43] Johansson PI Sølbeck S Genet G Stensballe J Ostrowski SR. Coagulopathy and hemostatic monitoring in cardiac surgery: an update. Scand Cardiovasc J (2012) 46(4):194–202. 10.3109/14017431.2012.671487 22375889

[44] Lisman T Porte RJ. Rebalanced hemostasis in patients with liver disease: evidence and clinical consequences. Blood (2010) 116(6):878–85. 10.1182/blood-2010-02-261891 20400681

[45] Clevenger B Mallett SV. Transfusion and coagulation management in liver transplantation. World J Gastroenterol (2014) 20(20):6146–58. 10.3748/wjg.v20.i20.6146 24876736 PMC4033453

[46] Wikkelsoe AJ Afshari A Wetterslev J Brok J Moeller AM. Monitoring patients at risk of massive transfusion with thrombelastography or thromboelastometry: a systematic review. Acta Anaesthesiol Scand (2011) 55(10):1174–89. 10.1111/j.1399-6576.2011.02534.x 22092122

[47] Weber CF Görlinger K Meininger D Herrmann E Bingold T Moritz A Point-of-care testing: a prospective, randomized clinical trial of efficacy in coagulopathic cardiac surgery patients. Anesthesiology (2012) 117(3):531–47. 10.1097/ALN.0b013e318264c644 22914710

[48] National Institute for Health and Care Excellence. Detecting, Managing and Monitoring Haemostasis: Viscoelastometric Point-of-Care Testing (ROTEM, TEG and Sonoclot Systems) [Internet]. (2014). Available from: http://www.nice.org.uk/guidance/dg13

[49] Solomon C Collis RE Collins PW. Haemostatic monitoring during postpartum haemorrhage and implications for management. Br J Anaesth (2012) 109(6):851–63. 10.1093/bja/aes361 23075633 PMC3498756

[50] Brenner T Schmidt K Delang M Mehrabi A Bruckner T Lichtenstern C Viscoelastic and aggregometric point-of-care testing in patients with septic shock – cross-links between inflammation and haemostasis. Acta Anaesthesiol Scand (2012) 56(10):1277–90. 10.1111/j.1399-6576.2012.02750.x 22897591

[51] Haase N Ostrowski SR Wetterslev J Lange T Møller MH Tousi H Thromboelastography in patients with severe sepsis: a prospective cohort study. Intensive Care Med (2015) 41(1):77–85. 10.1007/s00134-014-3552-9 25413378

[52] Müller MC Meijers JCM Vroom MB Juffermans NP. Utility of thromboelastography and/or thromboelastometry in adults with sepsis: a systematic review. Crit Care (2014) 18(1):R30. 10.1186/cc13721 24512650 PMC4056353

[53] Yang Y Yao Z Dai W Shi P Luo L Zhang C. Changes of thrombelastography in patients undergoing elective primary total knee and total hip replacement with low molecular heparin prophylaxis. J Orthop Surg Res (2014) 9:52. 10.1186/s13018-014-0052-0 24998166 PMC4100560

[54] Bai J Zheng QW Fu SH Li XX Li YR Zhou Y Association between thrombelastography system and thromboembolic and bleeding events in Chinese aged people. Int J Clin Exp Med (2013) 6(4):310–9. 23641310 PMC3631558

[55] Park MS Martini WZ Dubick MA Salinas J Butenas S Kheirabadi BS Thromboelastography as a better indicator of hypercoagulable state after injury than prothrombin time or activated partial thromboplastin time. J Trauma (2009) 67(2):266–75; discussion 275–6. 10.1097/TA.0b013e3181ae6f1c 19667878 PMC3415284

[56] Müller M Balvers K Binnekade JM Curry N Stanworth S Gaarder C Thromboelastometry and organ failure in trauma patients: a prospective cohort study. Crit Care (2014) 18(6):687. 10.1186/s13054-014-0687-6 25539910 PMC4305250

[57] Patel R Cook DJ Meade MO Griffith LE Mehta G Rocker GM Burden of illness in venous thromboembolism in critical care: a multicenter observational study. J Crit Care (2005) 20(4):341–7. 10.1016/j.jcrc.2005.09.014 16310605

[58] Eller T Busse J Dittrich M Flieder T Alban S Knabbe C Dabigatran, rivaroxaban, apixaban, argatroban and fondaparinux and their effects on coagulation POC and platelet function tests. Clin Chem Lab Med (2014) 52(6):835–44. 10.1515/cclm-2013-0936 24406289

